# An immune-humanized patient-derived xenograft model of estrogen-independent, hormone receptor positive metastatic breast cancer

**DOI:** 10.1186/s13058-021-01476-x

**Published:** 2021-10-30

**Authors:** Sandra D. Scherer, Alessandra I. Riggio, Fadi Haroun, Yoko S. DeRose, H. Atakan Ekiz, Maihi Fujita, Jennifer Toner, Ling Zhao, Zheqi Li, Steffi Oesterreich, Ahmed A. Samatar, Alana L. Welm

**Affiliations:** 1 Department of Oncological Sciences, University of Utah, 2000 Circle of Hope, Salt Lake City, UT 84112 USA; 2 Huntsman Cancer Institute, University of Utah, 2000 Circle of Hope, Salt Lake City, UT 84112 USA; 3 Department of Pharmacology and Chemical Biology, UPMC Hillman Cancer Center, Magee Women’s Research Institute, University of Pittsburgh, 204 Craft Avenue, Pittsburgh, PA 15213 USA; 4 Zentalis Pharmaceuticals, Inc., 10835 Road to the Cure, Suite 205, San Diego, CA 92121 USA

**Keywords:** ER+ metastatic breast cancer, ER+ PDX, Humanized PDX, Immune-humanization, *ESR1* mutation, Y537S, Endocrine resistance model, Estrogen supplementation, Breast cancer tumor microenvironment

## Abstract

**Background:**

Metastatic breast cancer (MBC) is incurable, with a 5-year survival rate of 28%. In the USA, more than 42,000 patients die from MBC every year. The most common type of breast cancer is estrogen receptor-positive (ER+), and more patients die from ER+ breast cancer than from any other subtype. ER+ tumors can be successfully treated with hormone therapy, but many tumors acquire endocrine resistance, at which point treatment options are limited. There is an urgent need for model systems that better represent human ER+ MBC in vivo, where tumors can metastasize. Patient-derived xenografts (PDX) made from MBC spontaneously metastasize, but the immunodeficient host is a caveat, given the known role of the immune system in tumor progression and response to therapy. Thus, we attempted to develop an immune-humanized PDX model of ER+ MBC.

**Methods:**

NSG-SGM3 mice were immune-humanized with CD34+ hematopoietic stem cells, followed by engraftment of human ER+ endocrine resistant MBC tumor fragments. Strategies for exogenous estrogen supplementation were compared, and immune-humanization in blood, bone marrow, spleen, and tumors was assessed by flow cytometry and tissue immunostaining. Characterization of the new model includes assessment of the human tumor microenvironment performed by immunostaining.

**Results:**

We describe the development of an immune-humanized PDX model of estrogen-independent endocrine resistant ER+ MBC. Importantly, our model harbors a naturally occurring *ESR1* mutation, and immune-humanization recapitulates the lymphocyte-excluded and myeloid-rich tumor microenvironment of human ER+ breast tumors.

**Conclusion:**

This model sets the stage for development of other clinically relevant models of human breast cancer and should allow future studies on mechanisms of endocrine resistance and tumor-immune interactions in an immune-humanized in vivo setting.

**Supplementary Information:**

The online version contains supplementary material available at 10.1186/s13058-021-01476-x.

## Background

Breast cancer is the second leading cause of cancer-related deaths in women worldwide [1]. With an estimated 276,480 newly diagnosed cases in the USA alone, breast cancer is the most frequently diagnosed malignancy in women. Between 25 and 30% of these breast cancer patients develop metastases, at which point the disease becomes incurable, and more than 42,000 patients die each year. Clinically, breast cancer is categorized into three major subtypes: (1) hormone receptor-positive, which refers to the presence of the estrogen receptor alpha (ER, encoded by the gene *ESR1*) and/or progesterone receptor (PR); (2) human epidermal growth factor receptor 2 (HER2)-positive, which refers to the overexpression of HER2 due to amplification of the *ERBB2* locus; and (3) triple negative breast cancer (TNBC), which lacks expression of the above-mentioned markers [2]. The majority of breast cancers are hormone receptor-positive, with nearly 75% expressing ER [1, 3]. While short-term survival rates of ER+ breast cancer patients are higher than in other subtypes, such as TNBC, more patients die from ER+ breast cancer than from any other subtype. Hence, there is an urgent need to discover and develop novel effective therapies for ER+ metastatic breast cancer (MBC).

Spreading of the tumor to distant organs, or metastasis, has fatal consequences and is the cause of most breast cancer deaths. While ER+ breast cancer can be treated with endocrine (hormone) therapy, metastatic tumors often develop endocrine resistance. The most commonly used endocrine therapies for ER+ MBC are selective estrogen receptor degraders (SERDs), selective estrogen receptor modulators, and aromatase inhibitors [4], with aromatases being key enzymes for the conversion of androgens into estrogens. Once ER+ tumors have metastasized and become endocrine resistant, effective treatments are limited. One established mechanism of endocrine resistance that limits treatment options is the acquisition of mutations in *ESR1*, which render the ER protein less dependent on estrogen for its function [5, 6]. *ESR1* mutations occur almost exclusively in metastatic disease that recurs after endocrine therapy, the most common *ESR1* mutations cause ER to retain an active conformation without estrogen binding [5–7]. In addition, breast cancers with *ESR1* mutations can gain a basal-like gene expression profile and enhanced M2-like macrophage immune activation that is associated with poor prognosis [8].

Reliable model systems that recapitulate patients’ disease are fundamental for drug development with the goal to achieve better translatability into the clinic. However, models of human ER+ MBC are limited. Human breast cancer cell lines including T47D [9], MCF7 [9], and MDA-MB-231 [10] have been extensively studied, and have been engineered to have *ESR1* mutations. Others have generated breast cancer cell lines from patient-derived xenograft (PDX) models [11]. In response to the need for models that better recapitulate patient tumors, represent the wide range of inter-patient heterogeneity, and are reflective of today’s treatment paradigms, however, several ER + PDX models have been developed [12–15]. PDX models resemble the patient’s disease on a genomic, molecular, and cellular level [12, 15–19], and are therefore valuable tools for research.

One caveat of PDX models is that they are grown in immunodeficient mouse strains, and therefore lack a functional immune system. It is well established that the tumor microenvironment is involved in various stages of solid tumor progression and metastasis [20–23] and, importantly, can influence treatment outcome and drug resistance [24–26]. In breast cancer, T cell infiltration is dynamic and heterogenous, and T cells are often excluded from tumors due to an immunosuppressive microenvironment, rendering immunotherapy largely ineffective [27]. On the other hand, myeloid cells robustly infiltrate breast tumors and are important for pro-tumorigenic effects [28]. Cells of the myeloid lineage, such as tumor-associated macrophages (TAMs), have been shown to play a fundamental role in disease progression. In breast cancer, the most dominant macrophage types are TAMs with a tumor-promoting “M2-like” phenotype [29], and high TAM infiltration correlates with poor prognosis [30, 31]. TAMs can negatively influence the efficacy of PARP inhibitors [24] and, in ER+ human breast cancer cell lines, TAMs have been implicated in resistance to endocrine therapy [32]. Taken together, these data underscore the importance of generating immune-humanized models for ER+ breast cancer to better define tumor-host interactions and improve patient outcome by uncovering strategies to make this disease more responsive to immunotherapy.

Immune-humanization of mice has been previously described [33], and such mice have been utilized in xenograft studies using human breast cancer cell lines [34–36] or TNBC PDXs [37]. However, immune-humanized ER+ PDXs have not been yet been reported. Thus, we sought to generate an immune-humanized PDX model of ER+ disease for which treatments are urgently needed: endocrine-resistant MBC with a naturally occurring *ESR1* mutation. Importantly, we found that immune-humanization of this breast cancer subtype recapitulates the lymphocyte-sparse and myeloid-rich immune milieu of certain human ER+ breast tumors: over 15% of luminal B breast cancers have been reported to display a lymphocyte-depleted milieu [38].

## Methods

### Experimental animals and health monitoring

All procedures were approved by the University of Utah Institutional Animal Care and Use Committee (IACUC). NSG mice (NOD.Cg-Prkdcscid Il2rgtm1Wjl/SzJ) or NSG-SGM3 mice (NOD.Cg-Prkdcscid Il2rgtm1Wjl Tg(CMV-IL3,CSF2,KITLG)1Eav/MloySzJ) were purchased from Jackson Laboratories one week before the beginning of an experiment and received 4100 ppm Uniprim Diet (3980X, 2% maltodextrin, TD cat. #200733) from Envigo upon arrival. Two days after arrival and throughout the entire experiment, mice received acidified drinking water and were supplemented with gel diet 76A (Clear H2O, cat. # 72-07-5022). During the first 3 weeks of an experiment and in cases of weight loss, mice also received peanut butter as a supplement (Spread The Love, cat. #B06ZXZ3JPZ) and some mice received oral antibiotic treatment with 19 mg/kg/day baytril Baytril/Enrosite (enrofloxacin). Health was monitored closely and mice weights were measured daily during the first three weeks of experiments and at least 3 times per week until the experiment ended. All procedures were performed in a sterile hood.

### Preparation of acidified and estrogen supplemented drinking water

To prepare acidified drinking water, the pH of sterile water was adjusted to 2.8–3.0, which was previously shown to prevent infections in experimental mice [39]. For estrogen (E2) supplementation, estrogen was added to acidified drinking water as described before [15] at a final concentration of 8 μg/ml. Estrogen water was prepared freshly on the day of usage and changed every 3–4 days. Estrogen plasma concentrations in NSG or NSG-SGM3 mice supplemented E2 supplemented acidified drinking water is shown in Additional file 1: Fig S1.

### Busulfan treatment

Busulfan (Selleck Chemicals, cat. #S1692) was reconstituted fresh at 49 mg/ml in DMSO immediately before each usage. For complete dissolving, busulfan solution was heated to 55 °C, sonicated for 20 min and kept warm at 42 °C on a heat block. Prior to injection, busulfan was diluted in warm PBS to reduce DMSO injection to a maximum of 4% per injection. For myeloablation, mice were intraperitoneally injected with 20 mg/kg busulfan on 2 consecutive days.

### Injection of human HSCs

CD34^+^ hematopoietic stem cells (HSCs) isolated from healthy donor bone marrow (BM) were purchased from AllCells. On the day of injection, cells were thawed in a 37 °C water bath and transferred into a 50 ml conical tube. 20 ml of warm culture media (DMEM/F12 supplemented with 10% fetal bovine serum, FBS) were slowly added dropwise into the cell suspension while gently shaking the tube. Cells were centrifuged (200   for 20 min at RT) and washed again in 20 ml of culture media. After centrifugation (200×*g* for 20 min at RT), cell pellet was resuspended in 0.5–1 ml of HBSS and cell concentration was determined using an automated cell counter (Countess II). Cells were diluted in HBSS accordingly and kept on ice until injection. 85,000 cells were intravenously (IV) injected into experimental mice in 100 ul HBSS using a 29 G × 12.7 mm (1/2″) needle (BD Biosciences, cat. # 324702).

### Implantation of breast PDX tumor tissue into mice

HCI-013 PDX tumor fragments were implanted into the inguinal mammary fat pad (MFP) of experimental mice as previously described [40]. Briefly, tumor fragments were thawed, washed three times in warm culture media (DMEM/F12 supplemented with 10% FBS) and kept on ice until implantation. Mice were anesthetized, the area of future incision was disinfected and PDX tumor fragments were implanted into the inguinal MFP. The fat pad posterior to the tumor implant including the area of the inguinal lymph node was cleared and the skin was closed with staples. Wound clips were removed 11–14 days post-surgery and tumors were measured twice a week using a digital caliper (9 mm, Braintree Scientific, Inc.). Tumor volume was calculated using the formula:

$${\text{Volume}}_{{{\text{Tumor}}}} = \left( {{\text{length}} \times {\text{width}}^{{2}} } \right)/{2}$$

### Estrogen pellet implantation and monitoring in vivo estrogen levels

Beeswax pellets containing 0.2 mg or 0.4 mg of E2 were prepared as published previously [15, 40]. To implant E2 pellets into mice, the mouse was anesthetized and prepared for surgery similar to the PDX tumor implantation protocol. After an incision was made into the skin, a dull surgical forcep was then used to introduce an E2 pellet subcutaneously, contralateral to the tumor implantation site between the skin and the peritoneal wall. The E2 pellet was inserted into the pocket and the wound was closed using wound staples. To monitor systemic estrogen levels, one blood drop was obtained from the submandibular vein and plasma was isolated by centrifugation (2000×*g*, 15 min, 4 °C). Estrogen concentrations were determined in the plasma using the Mesoscale Discovery (MSD) Custom Steroid Human Panel assay according to manufacturer’s instructions. Estrogen plasma concentrations of NSG or NSG-SGM3 mice implanted with E2 pellets is shown in Additional file 1: Fig. S1.

### Bilateral ovariectomy and generation of HCI-013EI

To generate HCI-013EI, 6–8 week old NSG mice were bilaterally ovariectomized (OVX) as previously described [15]. Briefly, one hour before surgery mice were given buprenorphine (0.1–0.2 mg/kg). Then, the day of surgery mice were anesthetized, two dorsal incisions were made and both ovaries were removed. PDX tumor fragments were implanted and another dose of buprenorphine (0.1–0.2 mg/kg) was given 8 h after the first dose. Mice were treated with carprofen (5 mg/kg) once a day for the following three days post-surgery to minimize pain. After harvesting the tumor, tumor cells were cultured for two weeks in phenol red-free HBEC medium [40] supplemented with charcoal-stripped FBS. Tumor cells were resuspended in 15 ul growth-factor reduced Matrigel per mouse, and re-implanted into the MFP in bilaterally-OVX NSG mice. No E2 supplementation was given.

### Tissue harvesting and processing

All studies were performed according to IACUC-approved procedures with veterinary supervision. Tumors were harvested before exceeding the approved maximum size of 3 cm for studies requiring immune reconstitution, or earlier in cases when the mouse health would be compromised. On the day of necropsy, mice were euthanized and blood was immediately drawn by cardiac puncture with a syringe that was filled with 50 ul of acid-citrate-dextrose (ACD)(Sigma Aldrich cat. #C3821). Blood was transferred into EDTA vacuum tubes (VACUETTE K2-EDTA, Greiner cat. #454052), which were also filled with 50 ul ACD, inverted twice and kept on ice. To isolate white blood cells, the blood was transferred into 15 ml conical tubes and red blood cells (RBCs) were lysed by adding 10 ml of 1 × RBC lysis buffer (Biolegend, cat. #420301) and incubating for 10 min at RT. Cells were spun down and RBC lysis was repeated if necessary. White blood cells were immediately stained for flow cytometry.

Tumors and MFPs were resected. Tumors were weighed and both tumors and MFPs were fixed in 10% NBF (neutral buffer formalin) for 24 h at 4 °C to generate formalin-fixed paraffin-embedded (FFPE) specimen.

Spleens were harvested and 1/5^th^ of the organ was fixed in 10% NBF 24 h at 4 °C to generate FFPE specimen. The remaining spleen was stored in DMEM/F12 media supplemented with 10% FBS and kept on ice. To generate a single cell suspension, spleens were placed onto a 70 µm cell strainer (Fisher Scientific, cat. #22363548) and mashed through it using the plunger of a 5 ml syringe (BD, cat. #309630). After rinsing the plunger and strainer with additional media, splenocytes were centrifuged (300×*g*, 5 min, 4 °C) and RBC lysis was performed on the cell pellet at least twice as described above. Splenocytes were filtered again through a 70 µm cell strainer and directly stained for flow cytometry. For BM isolations, femur and tibia were removed and carefully cleaned from soft tissue using a scalpel. Femurs and tibiae were separated and BM was isolated by either flushing or centrifugation. To flush out the BM, both ends of the femurs and tibiae were cut off, and a 1 ml syringe (BD, cat. #309659) with a 26G needle (BD, 26G × 3/8, 0.45 mm × 10 mm, cat. #305110) was used to scour the BM. To centrifugate BMs, the knees were removed from the leg bones by cutting right above and below the knee, as previously published [41]. Both tibia and femur were then placed into a 0.5 ml microtube with the bottom part cut off. The microtube containing the bones was then placed into a 1.5 ml microtube and centrifuged (≥ 10,000×*g*, 15 s, RT). Isolated BM was resuspended and carefully broken into single cells using a pipette. Then the cell suspension was filtered through a 70 µm cell strainer, RBC lysis was performed, and cells were stained for flow cytometry.

All FFPE tissues were fixed in 10% NBF for 24 h at 4 °C, washed three times in PBS for 5 min and placed in 70% ethanol at 4 °C until tissue processing and paraffin embedding were performed. For all studies involving PDX tumor bearing animals, mice were checked for metastases in other organs, such as lungs and liver. If present, axillary lymph nodes were harvested and fixed as FFPE specimen as described above.

### Flow cytometry

All flow cytometry analysis was performed using freshly isolated tissues and cells. Fc receptor blocking solution was prepared freshly by mixing a ratio of 10 μl/10^7^ cells of mouse Fc receptor blocking (Miltenyi Biotec, cat. #130-092-575) with 15 μl/10^7^ cells of human Fc receptor blocking (Miltenyi Biotec, cat. #130–059-901), then diluted in the same volume of FACS buffer (2% FBS in PBS). Cells were resuspended in 50 μl of Fc receptor blocking solution, transferred into a 96-well round-bottom plate (Greiner bio-one, cat. #650101) and incubated for 20 min on ice. The antibody cocktail was prepared by mixing the following antibodies: 0.5 μl/test BUV395 rat anti-mouse CD45 Clone 30-F11 (RUO) (BD Bioscience, cat. #564279), 5 μl/test APC mouse anti-human CD45 Clone HI30 (eBioscience, cat. #17-0459-42), 5 μl/test BV510 mouse anti-human CD11b/MAC-1 Clone ICRF44 (BD Bioscience, cat. #563088), 2.5 μl/test PE mouse anti-human CD56 (NCAM) Clone 5.1H11 (Biolegend, cat. #362508), 5 μl/test PerCP/Cy5.5 mouse anti-human CD3 Clone UCHT1 (Biolegend, cat. #300430), 5 μl/test FITC mouse anti-human CD19 Clone HIB19 (Biolegend, cat. #302206). The antibody mix of 23 μl/test was diluted with 27 μl/test FACS buffer. 50 μl of diluted antibody cocktail was added directly to each well and incubated for 30 min in the dark on ice. Unstained control wells received 50 μl of FACS buffer. After incubation, 100 μl FACS buffer was added to each well and the plate was centrifugated (300×*g*, 5 min, 4 °C). Cell pellets were washed once with 200 μl FACS buffer and once with 200 μl PBS. After the last centrifugation (300×*g*, 5 min, 4 °C), 100 μl of a 1:2000 dilution of Fixable Viability Dye eFluor® 780 (eBioscience, cat # 65-0865-18) in PBS was added to each well and incubated for 20 min in the dark. Unstained control wells received 100 μl of PBS. After incubation, 100 μl FACS buffer was added to each well and the plate was centrifuged (300×*g*, 5 min, 4 °C). Cell pellets were resuspended in 200 μl FACS buffer, transferred into a FACS tube (Olympus Plastics, cat. # 14-360) and stored at 4 °C with an aluminum foil cover until data acquisition.

Flow cytometry was performed immediately on freshly stained unfixed samples, and 0.5–1 million events/sample were acquired. Compensation was performed for each antibody individually using a 1:3 dilution of UltraComp eBeads™ Compensation Beads (Thermo Fisher, cat. # 01-2222-42) in PBS. 1 drop of the bead dilution was mixed with 1 μl of antibody and incubated for 20 min on ice in the dark. After centrifugation (600×*g*, 5 min, 4 °C), supernatant was discarded, beads were resuspended in 200 μl PBS and 5000 events were acquired. FlowJo version 10.5.3 was used for data analysis. All flow cytometry experiments included controls (FMOs,fluorescent minus one), unstained cell, and human peripheral blood mononuclear cells (PBMCs) mixed with NSG organs as positive controls for all human antibodies. Cells killed by repeated freeze/thaw cycles were used as a control for the viability staining. All gates were set based on FMO controls and the gating strategy which was applied to all samples is displayed in Additional file 1: Fig. S2.

### PDX tumor histology, IHC and IF stains

FFPE specimens were sectioned by the Biorepository and Molecular Pathology Shared Resource at Huntsman Cancer Institute. To obtain serial sections of tumors, 5 μm thick sections were collected, and hematoxylin–eosin (HE) staining was performed on sections every 35 μm to confirm tumor content and assess morphology.

All antibodies and immunohistochemistry (IHC)/immunofluorescence (IF) staining conditions used in this study are listed in Additional file 1: Fig. S3. Briefly, sections were incubated at 60 °C for 60 min, deparaffinized and rehydrated following standard procedures. Antigen retrieval was performed for 20 min in boiling buffer in the microwave, with the following exceptions. For PHH3 and CC3, the water bath was used at 60 °C ON, and for CAM5.2 ice-cold trypsin enzymatic antigen retrieval (abcam, cat. #ab970) was applied for 10 min at RT. For IHC (except for PHH3 and CC3), tissue slides were subsequently incubated in 3% H_2_0_2_ methanol buffer for 10 min at RT to block endogenous peroxidase activity. Tissue sections were then incubated in PBS blocking buffer containing 5% bovine serum albumin (Sigma-Aldrich, cat. #A7906), 10% normal goat serum (Jackson ImmunoResearch, cat. #005-000-121), 10% normal human serum (Jackson ImmunoResearch, cat. #009-000-121) and FcR blocking reagents for mouse (1:100, Miltenyi Biotech, cat. #130-092-575) and human (1:100, Miltenyi Biotech, cat. #130-059-901) for 1 h at RT. In addition, if mouse primary antibodies were used, the M.O.M Immunodetection kit (Vector Labs, cat. #BMK-2202) was added to the buffer. For double IHC and IF, the Avidin/Biotin (Vector Labs, cat. #SP-2001) and Streptavidin/Biotin (Vector Labs, cat. #SP-2002) blocking kits were used, respectively. For single IHC, staining was visualized with the 3,3-diaminobenzidine—Peroxidase substrate (Vector Labs, cat. #SK-4100), using Hematoxylin as a counterstain (Sigma-Aldrich, cat. #MSH32). For dual IHC, VECTASTAIN ABC kit—Peroxidase HRP (Vector Labs, cat. #PK-4000) and Vector AEC—Peroxidase substrate (Vector Labs, cat. #SK-42000) were used for CAM5.2 visualization, whereas VECTASTAIN ABC-AP kit—Alkaline Phosphatase (Vector Labs, cat. #AK-5000) and Vector Blue—Alkaline Phosphatase Substrate (Vector Labs, cat. #SK-5300) were used for PHH3 and CC3 visualization. For double IF, Fluorescent Streptavidin kit (Vector Labs, cat. #SA-1200) was used to amplify hCD45 signal. Single and double IF sections were counter-stained with the nuclear stain 4′, 6-diamidino-2-phenylindole dihydro-chloride (DAPI) for 20 min at RT. IHC stains were mounted with the Cytoseal Mounting Medium, Richard-Allan Scientific, Iow (60 s) (VWR, cat. #48212-154) and the VectaMount™ AQ Mounting Medium (Vector Labs, cat. #H-5501), respectively. IF stains were mounted using the VECTASHIELD Vibrance Antifade Mounting Medium (Vector Labs, cat. #H-1700). For each staining, secondary antibody controls were performed on serial sections.

### Slide digitalization and quantification

IF and IHC tissue slides were digitalized using a *Pannoramic MIDI II* (3DHISTECH Ltd., Budapest, Hungary) and an Axio Scan.Z1 (ZEISS, Jena, Germany) automatic slide scanners, respectively. Automated staining quantification was done using QuPath software v0.2.3 [42]. Briefly, after uploading the images and selecting for the appropriate image type (i.e. Brightfield: H-DAB for IHC or Fluorescence for IF) (Additional file 1: Fig. S4, step 1), the brightness/contrast tool was used on the entire image to improve the visibility of the stains. Subsequently, a small annotation was drawn (Additional file 1: Fig. S4, step 2) to train the software for positive cell detection by editing the appropriate parameters based on the cellular location and the intensity of each stain (Additional file 1: Fig. S4, step 3). Following the creation of a region of interest (ROI) encompassing the tumor and excluding the surrounding MFP (Additional file 1: Fig. S4, step 4), the improved positive cell detection analysis was finally applied to the ROI. Extra-tumoral tissue was excluded from the analysis in order to capture intratumoral immune cells only. Snapshots and images were taken with the CaseViewer software (2.4.0.119028). For each stain, no visible signal was detected in the secondary antibody control slides.

### Droplet digital PCR to identify ESR1 mutations

Droplet digital PCR (ddPCR) was performed as previously published [15, 43] to identify potential *ESR1* hotspot mutations in HCI-013 and HCI-013EI tumors. Specifically, RLT buffer (Qiagen, cat. # 1053393) containing beta-mercaptoethanol was added to tumor tissues which were then homogenized on ice. Immediately after, the QiagenAll Prep kit (Qiagen, cat. # 80204) was used to isolate genomic DNA (gDNA) and total RNA following manufacturer's instructions. After cDNA synthesis using PrimeScript RT Reagent Kit (Takara, RR037), 50 ng gDNA or cDNA was used as templates and primer/probe sets for four specific *ESR1* mutations (Y537S/Y537C/Y537N/D538G) was added [43]. Droplets were generated using the Bio-Rad QX200 AutoDG Droplet Digital PCR system, followed by an amplification step of the *ESR1* ligand binding domain fragment using a thermo cycler. Wildtype (WT) and mutant probes were detected with a Bio-Rad QX200 droplet reader and the allele frequency of mutant *ESR1* was calculated using the QuantaSoft software version 1.7. To ensure calls were made correctly, droplets containing a positive control (gDNA from genome-edited Y537S *ESR1* mutant MCF7 cell line) or negative control (gDNA from *ESR1* WT MCF7 cell line), and background control (ddH_2_O) were included.

### AAVs for delivery of human cytokines

Adeno-associated virus 9 (AAV9) vectors encoding human interleukin-7 (IL7), IL15, thrombopoietin, or empty vector controls were purchased from Vector Biosystems Inc, diluted in PBS and IV injected into mice at the following concentrations: 1.0 × 10^11^ gc/ml AAV9-CAG-h-IL7-WPRE; 2.0 × 10^11^ gc/ml AAV9-CAG-h-IL15-WPRE; 1.0 × 10^10^ gc/ml AAV9-CAG-h-THPO-WPRE.

## Results

To develop an immune-humanized ER+ model representing a major unmet medical need in breast cancer, we chose the HCI-013 PDX line, which is a metastatic, endocrine resistant ER+ model of lobular breast cancer [15]. Importantly, this model possesses a naturally- occurring Y537S hotspot mutation in the ligand binding domain of *ESR1*, which represents one of the two most common *ESR1* mutations in MBC. For immune-humanization experiments, we chose NSG-SGM3 mice, an immunodeficient strain with genes knocked-in to express human stem cell factor (SCF), granulocyte–macrophage colony stimulating factor (GM-CSF) and IL-3 [44]. The expression of these three human cytokines enables improved myeloid immune reconstitution in mice [44–46].

### Optimization of estrogen supplementation in HCI-013 PDX tumor growth for immune-humanization

**Fig. 1:**
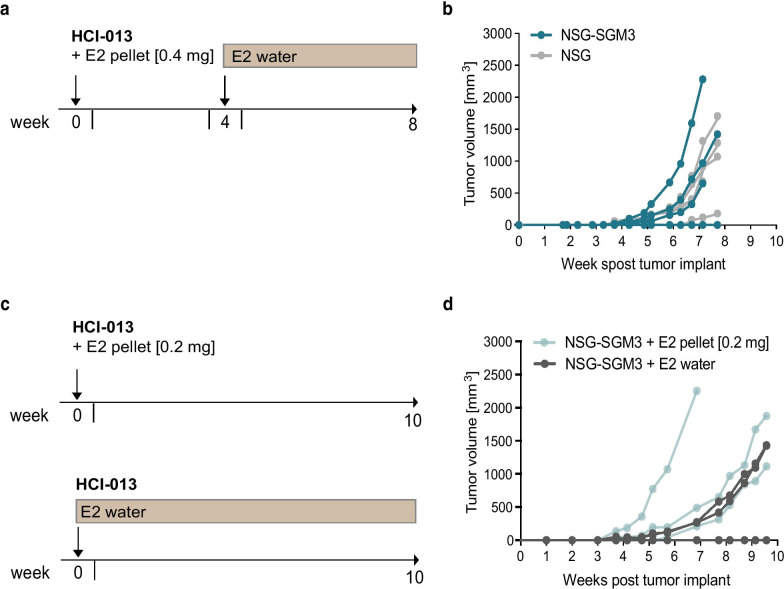
Optimization of exogenous estrogen supplementation methods to promote HCI-013 tumor growth in experimental mice. **a** Experimental timeline. 8 weeks old female NSG-SGM3 or NSG mice were inoculated with HCI-013 tumor fragments into the MFP (*n* = 5). On the same day, mice were implanted with 0.4 mg E2 pellets, while 4 weeks post tumor implantation, mice received E2 supplemented acidified drinking water until the end of the experiment. This reflects our standard exogenous estrogen supplementation for ER+ PDX lines [15]. **b** Tumor growth was monitored until mice were harvested 8 weeks post tumor implantation (*n* = 5). HCI-013 PDX tumors have previously been reported to have heterogeneous growth (DeRose et al. 2011), which we also observed here. **c** Experimental timeline. 8 weeks old female NSG-SGM3 mice were implanted with HCI-013 tumor fragments into the MFP (*n* = 5). On the same day, mice were either implanted with 0.2 mg E2 pellets, which represents half of the pellet size of our standard protocol (upper timeline), or received E2 supplemented acidified drinking water until the end of the experiment (lower timeline). **d** Tumor growth was monitored until mice were harvested 10 weeks post tumor implantation (*n* = 5)

One of the challenges in modeling human ER+ breast cancer in mice is the typical requirement for supplemental estrogen due to low levels of endogenous murine E2 [47, 48]. HCI-013 is an E2-responsive model [49] and, in the presence of exogenous E2, HCI-013 reliably produces tumors in NSG mice, with somewhat heterogeneous growth rates [15]. Since the majority of our ER+ PDX models, including HCI-013, were developed in NSG hosts, we first asked whether the expression of human cytokines in NSG-SGM3 mice altered the growth of HCI-013 PDX tumors. We therefore implanted HCI-013 tumors in NSG and NSG-SGM3 mice and tested our standard protocol for estrogen supplementation [15] using a 0.4 mg E2 pellet implanted at the time of surgery, followed by the addition of E2 to the drinking water 4 weeks post tumor inoculation (Fig. 1a). Tumors grew comparably in both mouse strains, usually reaching 1000 mm^3^ in approximately 7–8 weeks, thus ruling out the effect of human cytokines on tumor growth (Fig. 1b). However, this relatively fast tumor growth would not allow for complete immune reconstitution in NSG-SGM3 mice, which takes a minimum of 9 weeks for myeloid cell development and longer time for T cell and NK cell development [44, 50–52]. Thus, we next tested several conditions with reduced estrogen doses in NSG-SGM3 mice to find a setting in which tumors would grow well, but slowly enough to facilitate the time required for immune system development. For this, we supplemented exogenous estrogen by either implanting half dose (0.2 mg) E2 pellets or giving E2 only in the drinking water (Fig. 1c). We found that both strategies supported a tumor growth rate that gives enough time for immune humanization (Fig. 1d). We decided to move forward with E2 supplementation via drinking water, since it is a non-invasive method and enables estrogen delivery to be stopped at any time by switching back to acidified drinking water.

### Effects of supplemental estrogen on immune humanization

**Fig. 2:**
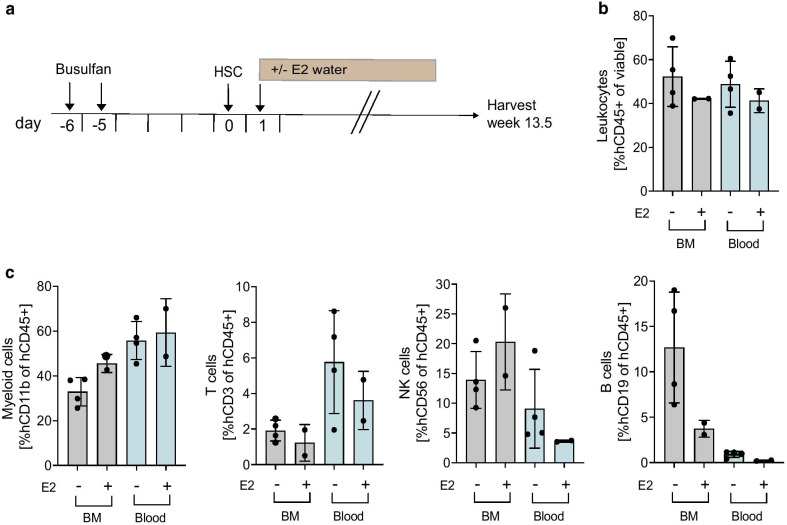
Effect of E2 supplementation on immune humanization in mice. **a** Experimental timeline. 6–7 weeks old NSG-SGM3 female mice were injected with 20 mg/kg/day busulfan on two consecutive days. Five days later, mice were IV injected with 85,000 hCD34^+^ HSCs and 24 h later received E2 supplemented acidified drinking water for 3.5 weeks (*n* = 5, HSC donor ID 7734, KIR-mismatched to HCI-013). Control mice were kept on unsupplemented acidified drinking water. Mice were harvested 13.5 weeks post-humanization, at which point BM and blood were freshly isolated, and flow staining was performed. Flow cytometry was performed on the day of tissue harvest without fixation. **b** Flow cytometry analysis of hCD45^+^ cells in BM, and blood of humanized mice receiving E2 or unsupplemented acidified drinking water are shown as percent of viable cells (*n* = 2–4). **c** Flow cytometry analysis of hCD11b^+^, hCD3^+^, hCD56^+^ and hCD19^+^ cells in BM and blood of humanized mice receiving E2 or unsupplemented acidified drinking water are shown as percent of hCD45^+^ cells (*n* = 2–4)

Estrogen can have profound effects on the immune system [53, 54], so we asked whether supplementation of estrogen in the drinking water would affect reconstitution of the human immune system in NSG-SGM3 mice. To test this, we first performed an experiment to humanize NSG-SGM3 mice without implantation of PDX tumors (Fig. 2). For immune-humanization, NSG-SGM3 mice were treated with busulfan to achieve myeloablation as previously described [55, 56]. We chose this approach over irradiation since the latter might be more prone to cause adverse effects, including the severe anemia reported in irradiated humanized MISTRG and NSG-SGM3 mice [57, 58]. Mice were then immune-humanized by IV injection of human BM-derived CD34+ HSCs (Fig. 2a). It has been previously reported that HLA-matching donor HSCs to PDX tumor does not affect tumor growth [33, 59]. However, to minimize chances of an allograft reaction while maximizing chances of immune T cell tolerance against human PDX tumors, we implanted the tumors one day after IV injection of HSCs, so that newly generated T cells could develop in parallel with a growing tumor. BM-derived immature HSCs were used instead of mature PBMCs to avoid graft-versus-host disease (GvHD) [33]. The acidified drinking water was supplemented with E2 beginning 24 h post-humanization, which is the time point where we planned to implant PDX tumors for future experiments. Compared to the control group, which received acidified drinking water without E2, no differences in humanization were observed in BM or blood based on the percentage of hCD45+ cells, which averaged 46.3% of the mouse blood across all experimental groups (Fig. 2b). As a reference, commercially available humanized mice are considered to be humanized at minimum ~ 25% hCD45+ cells in mouse blood. In addition, no significant effects of E2 on the development of myeloid (hCD11b+) cells, T (hCD3+) cells, NK (hCD56+) cells or B (hCD19+) cells were observed, although there was a trend towards development of more myeloid cells, and fewer B and T cells in mice receiving E2 (Fig. 2c).

**Fig. 3:**
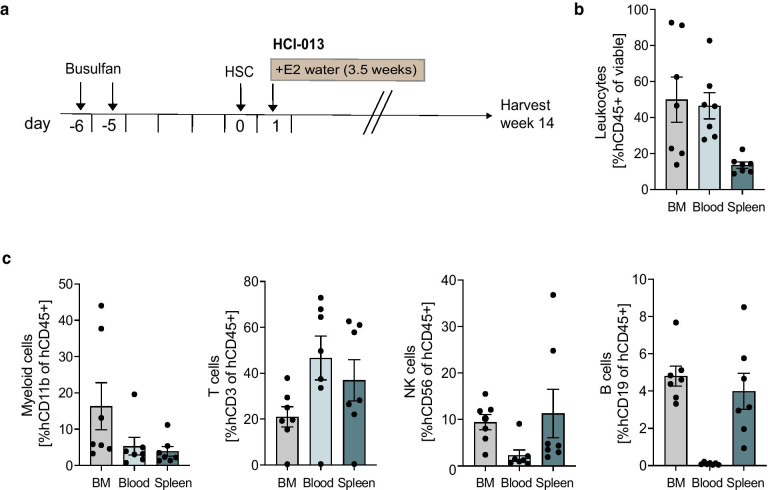
Exogenous E2 supplementation in immune-humanized mice bearing HCI-013 tumors. **a** Experimental timeline. 6–7 weeks old female NSG-SGM3 mice were injected with 20 mg/kg/day busulfan on two consecutive days. Five days later, mice were IV injected with 85,000 hCD34^+^ HSCs and 24 h later implanted with HCI-013 tumor fragments into the MFP. Animals received E2 supplemented drinking water for 3.5 weeks until the end of the experiment (*n* = 10, HSC donor ID 12241, KIR-matched to HCI-013). Mice were harvested 14 weeks post-humanization. Control tumors were implanted into non-humanized NSG-SGM3 mice (*n* = 5). On the day of harvest, BM, blood and spleens were isolated, and flow staining and flow cytometry were performed on the same day without fixation. **b** Flow cytometry analysis of hCD45^+^ cells in BM, blood and spleen of humanized mice are shown as percent of viable cells (*n* = 7). **c** Flow cytometry analysis of hCD3^+^, hCD11b^+^, hCD19^+^ and hCD56^+^ cells in BM, blood and spleens of humanized mice are shown as percent of hCD45^+^ cells (*n* = 7). All data shown as mean ± SEM

Unfortunately, we noticed that humanized mice exposed to exogenous E2 developed severe anemia after 1.5 weeks of estrogen supplementation, sometimes to the point of sickness that required euthanasia. Thus, we next investigated whether this was a reproducible phenomenon or an underlying donor-specific effect. We repeated the experiment using a different HSC donor and included HCI-013 tumor engraftment with E2 supplementation. After myeloablation, NSG-SGM3 mice were injected with HSCs, followed one day later by orthotopic implantation of HCI-013 PDX tumor tissue and E2 supplementation via drinking water (Fig. 3a). We chose the timing of PDX tumor implantation to be close to the injection of HSCs to avoid potential GvHD or graft-versus-tumor reactions by mature T cells in already-humanized mice. This way, T cells develop in tumor-bearing mice in the presence of tumor antigens, thus increasing the probability of T cell tolerance against human tumor cells. 14 weeks post-humanization, we found effective hCD45+ immune cell reconstitution in BM (~ 50%), blood (~ 47%), and spleen (~ 18%), and we were again able to detect the development of human myeloid cells, T cells, NK cells and B cells in each of these organs (Fig. 3b, c).

However, we again found that immune-humanized mice receiving E2 became severely anemic, thus excluding the possibility of a donor-specific effect given that HSCs were obtained from two different donors for these experiments. While tumors were palpable at the beginning of the experiment (Fig. 3), some mice had to be euthanized due to anemia prior to the planned endpoint. Removal of E2 from the drinking water reversed anemia in surviving mice, allowing the recovery of some animals (Additional file 1: Fig. S5), but tumor growth was not sustained, most likely due to the fact that E2 supplementation had to be stopped. Regarding the onset of anemia, we excluded the possibility of a donor-specific effect given that HSCs obtained from two different donors were used in these experiments. Since anemia also occurred in control mice that did not receive tumor implants, we concluded that anemia was not dependent on the presence of tumor. Instead, it appeared that even low levels of supplemental E2 delivered in drinking water caused severe anemia specifically in immune-humanized mice.

### Endogenous mouse estrogen levels are not sufficient for HCI-013 tumor growth

**Fig. 4:**
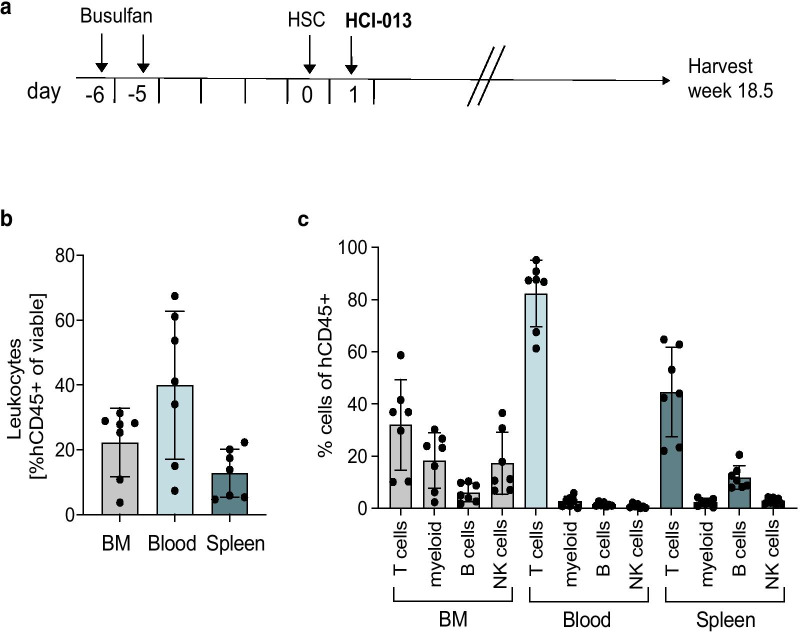
Immune humanization of mice bearing HCI-013 PDX tumors without exogenous E2. **a** Experimental timeline. 6–7 weeks old female NSG-SGM3 mice were injected with 20 mg/kg/day busulfan on two consecutive days. Five days later, mice were IV injected with 85,000 hCD34^+^ HSCs and 24 h later implanted with HCI-013 tumor fragments into the MFP. Animals were harvested 18.5 weeks post-humanization (*n* = 10, HSC donor ID BM4410, KIR-matched to HCI-013). Control tumors were implanted into non-humanized NSG-SGM3 mice (*n* = 3). On the day of harvest, BM, blood and spleens were isolated, and flow staining and flow cytometry were performed on the same day without fixation. **b** Flow cytometry analysis of hCD45^+^ cells in BM, blood and spleen of humanized mice are shown as percent of viable cells (*n* = 8). **c** Flow cytometry analysis of hCD3^+^, hCD11b^+^, hCD19^+^ and hCD56^+^ cells in BM, blood and spleens of humanized mice are shown as percent of hCD45^+^ cells (*n* = 8). All data are shown as mean ± SEM

Although the HCI-013 PDX model was originally developed in mice with exogenous E2 supplementation, we hypothesized that the presence of the Y537S mutation in *ESR1* might render the tumor independent of estrogen. We next tested whether HCI-013 could grow in immune-humanized mice without E2 supplementation, but with intact ovaries, leaving the PDX tumor exposed to low levels of endogenous murine estrogen. Thus, NSG-SGM3 mice with intact ovaries were immune-humanized followed by orthotopic implantation of HCI-013 PDX tumors without supplemental E2 (Fig. 4a). In this experiment, without E2 supplementation, mice did not become anemic, and tissues were collected 18.5 weeks post-humanization for human immune cell characterization. The results revealed typical reconstitution of the human immune system, as indicated by the presence of hCD45+ cells in the BM, blood, and spleen (Fig. 4b), and CD34+ HSCs differentiated into all of the major immune lineages including T cells, myeloid cells, B cells, and NK cells (Fig. 4c). At this later time point, T cells were the predominant human immune cells in all analyzed tissues. Unfortunately, though, in the absence of exogenous E2, no tumor growth was observed in either immune-humanized or non-humanized NSG-SGM3 mice. Thus, we concluded that endogenous estrogen levels from murine ovaries were not sufficient to support HCI-013 tumor growth in the time frame of our experiment, despite the presence of the Y537S mutation. This challenge, along with E2-induced anemia in immune-humanized mice, precluded us from successfully developing an immune-humanized PDX model of ER+ breast cancer that depends on supplemental E2.

### Development of an estrogen-independent HCI-013 PDX model

**Fig. 5:**
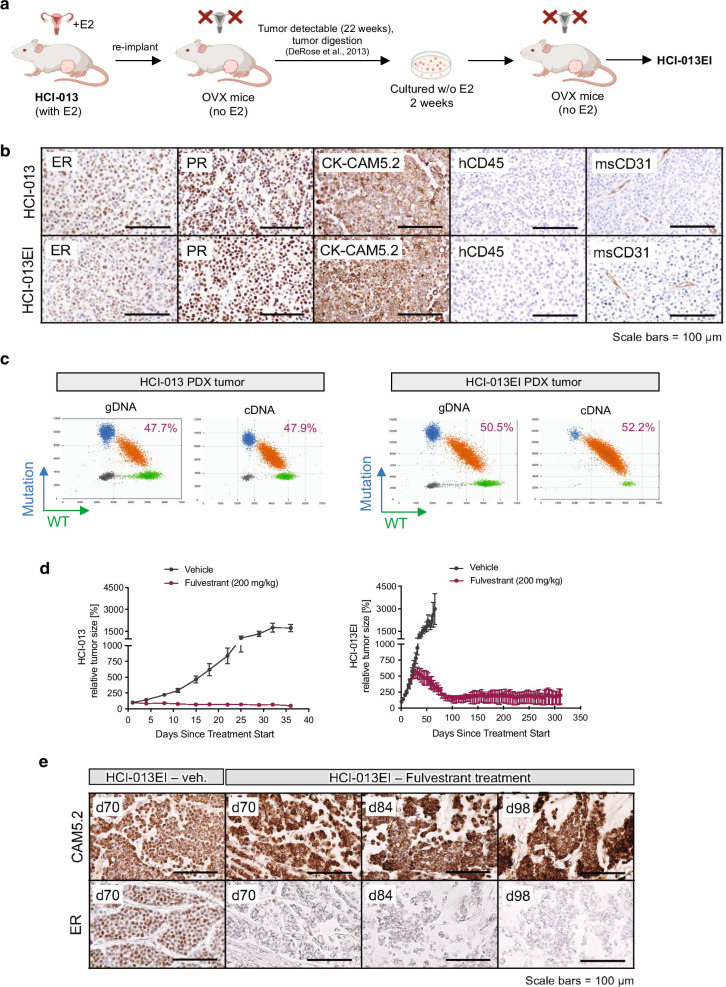
Development of an estrogen-independent HCI-013 PDX line (HCI-013EI). **a** HCI-013 PDX tumors were grown in mice with intact ovaries and exogenous E2 supplementation (0.4 mg E2 pellet, and E2 water starting 4 weeks post tumor implant). At end point, tumors were harvested and re-implanted into OVX mice with cleared fat pads, without E2 supplementation. Once fully grown, tumors were harvested, digested, and tumor cells were expanded in tissue culture for 2 weeks in estrogen deprived conditions (phenol red-free medium, with charcoal stripped serum and without estrogen supplementation). Tumor cells were then re-implanted into OVX mice with cleared fat pads, without E2 supplementation. The estrogen-independent tumors that grew out under these conditions were expanded, validated and named HCI-013EI. **b** Histological comparison of ER, PR, CAM5.2, hCD45, and mouse (ms) CD31 stains in HCI-013 and HCI-013EI PDX tumors. **c** Detection of Y537S homozygous ESR1 mutation in HCI-013 and HCI-013EI PDX tumors using droplet digital PCR. Graph shows 2D scatter plots with fluorescent detection of individual droplets with either gDNA or cDNA. Dots represent droplets with WT (green), or mutant *ESR1* (blue) genotypes, or droplets containing both WT and mutant *ESR1* (orange). Droplets without DNA content are indicated in black. **d** Tumor growth curves of HCI-013 and HCI-013EI under fulvestrant treatment (200 mg/kg). All data shown as mean ± SEM. **e** Histological comparison of ER and CAM5.2 IHCs in HCI-013EI PDX tumors treated with long-term fulvestrant or vehicle

After discovering the need to generate an estrogen-independent PDX model of ER+ MBC, we set out to develop a model of ER+ MBC that does not require E2 supplementation. To achieve this, we transplanted HCI-013 into OVX mice in the absence of exogenous E2 (Fig. 5a). Tumors eventually grew, albeit much more slowly than in mice receiving exogenous E2 [15]. We harvested these tumors, expanded them for two weeks as primary cultures in charcoal-stripped serum and phenol red-free medium, and then re-implanted them into OVX mice to validate their estrogen independence [15]. The resulting isogenic estrogen-independent HCI-013 PDX line (HCI-013EI) retains expression of ER and PR and resembles the morphology of the original line (Fig. 5b). Successful development of an estrogen-independent subline of HCI-013 was consistent with the fact that the patient’s tumor was resistant to multiple lines of endocrine therapy, and she unfortunately died from MBC. Interestingly, the allele frequency of the Y537S mutation in HCI-013EI PDX tumors remained similar to parental HCI-013 PDX tumors (Fig. 5c), suggesting that the ability of HCI-013EI to grow without exogenous E2 cannot simply be explained by an increase in mutational burden of *ESR1.*

We validated the endocrine resistant nature of HCI-013EI by treating with fulvestrant, which is a SERD that can be effective against mutant ER [60]. In parental HCI-013 PDX tumors, fulvestrant treatment led to immediate control of tumor growth (stable disease), but did not result in tumor regression (Fig. 5d). On the other hand, HCI-013EI PDX tumors did not initially respond to fulvestrant. Instead, they displayed a delayed response to the drug after 5–6 weeks of treatment. Long-term treatment with fulvestrant (for more than 300 days) was able to control tumor growth, but was insufficient to achieve a complete response in this model. Residual tumors, as expected, had low levels of ER following long-term fulvestrant treatment (Fig. 5e). Altogether, these data indicate that HCI-013EI tumors have reduced dependence on E2, and reflect a scenario of endocrine resistance, thus making it a good model for ER+ MBCs that are difficult to treat.

### Immune-humanization of the estrogen-independent HCI-013EI PDX line

**Fig. 6:**
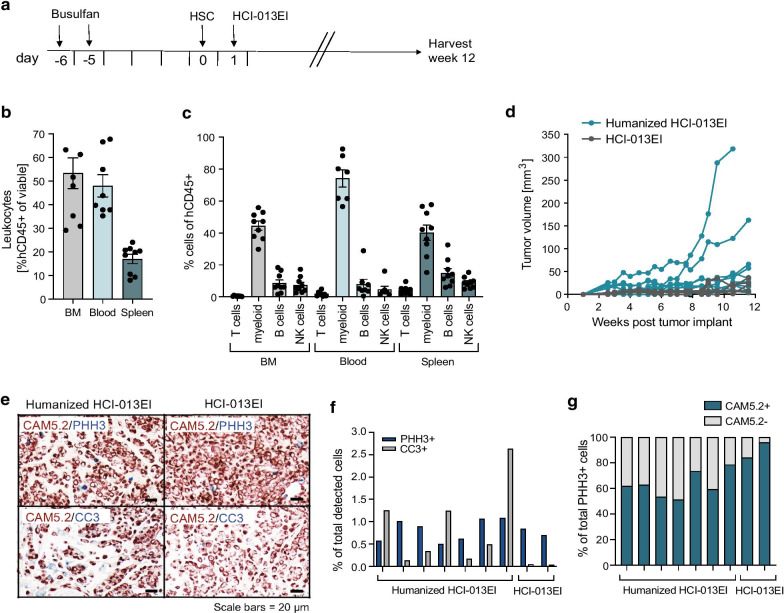
Immune humanization of mice bearing HCI-013EI PDX tumors. **a** Experimental timeline. 6–7 weeks old NSG-SGM3 female mice were injected with 20 mg/kg/day busulfan on two consecutive days. Five days later, mice were IV injected with 85,000 hCD34^+^ HSCs and 24 h later implanted with HCI-013EI tumor fragments into the MFP, without exogenous E2 supplementation. Animals were harvested 12 weeks post-humanization (*n* = 10, HSC donor ID 7734, KIR-mismatched to HCI-013). **b** Flow cytometry of hCD45^+^ cells in BM, blood and spleen of humanized mice are shown as percent of viable cells (*n* = 9). **c** Flow cytometry of hCD11b^+^, hCD3^+^, hCD56^+^ and hCD19^+^ cells in BM, blood and spleens of humanized mice are shown as percent of hCD45^+^ cells (*n* = 9). **d** HCI-013EI tumor growth of humanized mice compared to non-humanized control mice (humanized *n* = 9, controls, *n* = 5). **e** Representative images of double IHC stains of HCI-013EI PDX tumors from humanized and control mice. Upper panel: CAM5.2 (red) and PHH3 (blue). Lower panel: CAM5.2 (red) and CC3 (blue). **f** Quantification of **e** showing PHH3+ or CC3+ cells as percentages of total cells detected in HCI-013EI PDX tumors across both groups. **g** Quantification of **e** showing CAM5.2+ and CAM5.2− cells as percentages of all proliferating PHH3+ cells detected in HCI-013EI PDX tumors across both groups. All data shown as mean ± SEM

Next, we immune-humanized the HCI-013EI PDX model using a similar protocol for immune reconstitution as in previous experiments, but without the addition of E2 (Fig. 6a). At the endpoint, which was 12 weeks post-humanization, we again saw effective humanization in BM (~ 52%), blood (~ 47%), and spleen (~ 18%) (Fig. 6b), and confirmed with flow cytometry that myeloid cells, T cells, NK cells and B cells had differentiated in these mice (Fig. 6c). Interestingly, amongst the analyzed subpopulations myeloid cells were the predominant human cell type that developed in immune-humanized HCI-013EI PDXs. HCI-013EI tumors grew similarly in both humanized and non-humanized NSG-SGM3 mice, although there were two fast-growing outliers in the humanized group (Fig. 6d). Tumor take rate in humanized mice was 80% (*n* = 8/10 mice), and 60% in non-humanized mice (*n* = 3/5 mice), indicating that the reconstituted human immune cells did not reject PDX tumor growth. Double IHC stains of human-specific cytokeratin proteins (CAM5.2) and the proliferation marker phospho-histone H3 (PHH3) revealed that the fraction of proliferating tumor cells was similar between non-humanized and humanized mice, averaging 0.7% of cells in mitosis (Fig. 6e, f). Further analysis of PHH3+ cells revealed that most of the actively proliferating cells were human tumor cells in both humanized and non-humanized HCI-013EI tumors (Fig. 6g). Interestingly, while it was rare to find cleaved caspase 3 (CC3)-positive cells in non-humanized HCI-013EI tumors, a variable level of CC3 staining was detected in humanized tumors, suggesting that immune-humanization may contribute to apoptosis (Fig. 6f). However, we were able to successfully reconstitute human immune cells in an endocrine-resistant ER+ model of MBC, and immune-humanization did not result in rejection of the PDX tumors.

### Characterization of the tumor microenvironment in immune-humanized HCI-013EI PDX mice

**Fig. 7:**
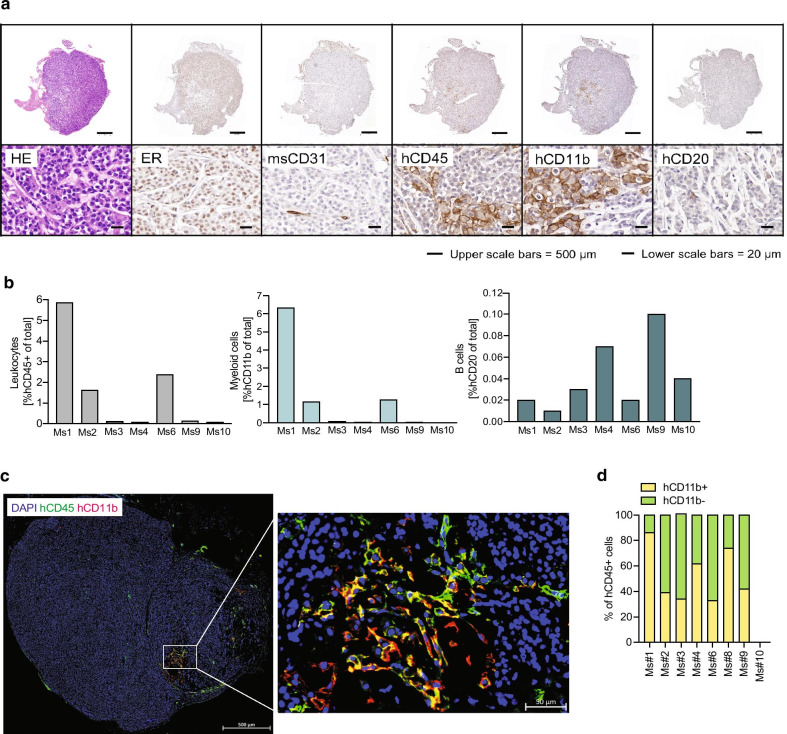
Tumor microenvironment of immune-humanized HCI-013EI PDX models. **a** Representative images showing histology of HE, ER, msCD31, hCD45, hCD11b and hCD20 stains in immune-humanized HCI-013EI PDX tumors from Fig. 6. **b** Quantification of **a** of all humanized mice showing intratumoral hCD45^+^ cells (left), hCD11b+ cells (middle), and hCD20^+^ cells (right) as percentages of total detected cells. **c** Representative image of double IF staining of the same tumors as in **a** showing hCD45 (green), and hCD11b+ cells (red). **d** Quantification of **e** showing hCD11b+ and hCD11b− cells as percentages of total hCD45+ cells detected in HCI-013EI PDX tumors of humanized mice. Note that the presence of tumor-infiltrating human myeloid cells was often found to localize within the core of HCI-013EI tumors

In an attempt to further dissect the nature of the reconstituted intratumoral human immune subpopulations, we characterized the tumor microenvironment of humanized HCI-013EI PDX tumors using IHC and IF techniques (Fig. 7). IHC staining showed ER expression in tumor cells is maintained in immune-humanized HCI-013EI PDXs (Fig. 7a). We also found that intratumoral blood vessels are of murine origin, similar to non-humanized PDX models [9], as tumors stained negative for hCD31 (data not shown). Whenever present, hCD45+ cells were distributed throughout the tumors (Fig. 7a). However, the degree of human immune cell infiltration varied greatly between animals (Fig. 7b), with some tumors displaying little or no detectable intratumoral human immune cells, despite good systemic immune reconstitution in all mice (Fig. 6b, c). Of note, double IF stains revealed that a large portion of tumor-infiltrating human immune cells are of the myeloid lineage (Fig. 7c, d), in line with myeloid (hCD11b+) cells being the predominant reconstituted subpopulation of all hCD45+ cells analyzed at this timepoint (Fig. 6c). Albeit rarely, intratumoral human B cells were also observed in some mice. On the contrary, we did not detect any human T cells or NK cells within HCI-013EI PDX tumors, although these cells were both reconstituted and each represented ~ 10% of the human immune cells detected within the spleen.

## Discussion

We report for the first time the development of an immune-humanized PDX model of endocrine resistant ER+ MBC that harbors a naturally occurring *ESR1* Y537S mutation. Y537S has been shown to be the second most frequently occurring *ESR1* mutation in endocrine resistant breast tumors in patients and mutated *ESR1* leads to aberrantly increased ER transcriptional activity [5–7]. To date, there are no treatments available that specifically target mutant ER*.* This is of high relevance since patients whose tumors harbour *ESR1* mutations have poor prognosis compared to patients whose tumors are WT *ESR1* [8]. Efforts have been made to generate valuable PDX models for ER^+^ MBC, but the need to use immunodeficient hosts to grow PDXs makes it impossible to study the known role of the immune system in tumor progression and treatment response. Hence, we set out to use a novel approach for human immune reconstitution in our model. One of the major differences between our humanization approach and other humanized models is that our tumors are inoculated into mice 24 h after HSC injection. This enables human immune cells to be exposed to tumor antigens while they are developing in the mouse. Immune-humanization had no negative effect on tumor growth in our study (Fig. 6d), and we did not find a correlation between the degree of immune reconstitution and tumor growth rates, which are naturally heterogeneous in PDX models. We suggest that this strategy might reduce the risk of allogenic T cell responses, while also avoiding GvHD that could occur if patient-matched mature T cells are used for immune reconstitution.

Characterization of the tumor microenvironment of our immune-humanized model showed a predominant myeloid cell infiltration into tumors. CD11b+ myeloid cells are one of the most abundant immune cell component of the human breast tumor microenvironment, where they have been reported to interact with other factors and various tumor cells to foster cancer progression and metastasis [61, 62]. Specifically, TAMs, myeloid-derived suppressor cells and dendritic cells have been shown to play a crucial role in tumor immune evasion by secreting IL-10, IL-12 and arginase-1, leading to suppression of CD8+ T-cell activity and reduced activity of effector T cells [63, 64]. Interestingly, the receptor tyrosine kinase RON, which is expressed on resident macrophages, has been recently shown to promote endocrine therapy resistance in *ESR1* mutated breast cancers [65]. Thus, since most of the tumor-infiltrating cells are of myeloid origin, our immune-humanized model would be an interesting system to study this mechanism further.

While we do detect human T cells (hCD3+) in peripheral blood and spleens of immune-humanized HCI-013EI PDX mice, T cells were not observed in the tumor microenvironment. Based on data from immune-humanized mice employing the use of HSCs, myeloid cells were shown to develop 8–9 weeks post-humanization, B cells develop after 10–11 weeks, while T cells develop after 1112 weeks, depending on the model used [66, 67]. Accordingly, in experiments that lasted 14 or 18.5 weeks post-humanization, we detected more T cells in blood, BM and spleen than we did at 12 weeks post-humanization. Hence, one explanation for our observation could be that at the 12-week timepoint, when we needed to end the experiment with the HCI-013EI model due to tumor burden, T cells had developed but had not yet started to infiltrate the tumors. Alternatively, since we used NSG-SGM3 mice, which enable optimal myeloid immune reconstitution in mice [44–46], it is possible that the myeloid cells restrict T cell recruitment into tumors, similar to what is seen in human breast cancers [28]. Yet another possibility for lack of intratumoral T cells in our model is that HCI-013EI might represent a “cold” tumor. Cold tumors (i.e. noninflamed) are described as solid cancers that have low immune cell infiltration, including low or absent numbers of CD3+ and CD8+ T cells [68–70]. Many breast tumors are considered to be immune “cold” [38], and this is one of the reasons why immune checkpoint blockade is not very effective against this disease [71]. In the case of HCI-013, it is impossible to know whether the original patient tumor was of the immune “cold” nature because no primary tumor material is available; the model was generated from a metastatic pleural effusion fluid sample.

Multiple efforts have been made to find ways to manipulate and improve the infiltration of T cells into the tumor. In one of these recent studies, vitamin D was shown to increase intratumoral T cells in a breast cancer model [72]. In another study in melanoma patients, injection of oncolytic viruses led to an increase in CD8+ T cell tumor infiltration, thereby promoting the efficacy of anti-PD1 immunotherapy [73, 74]. One possible application for the model we describe here would be to test novel strategies to increase T cell infiltration into the tumors as a way to increase susceptibility to immunotherapy for breast cancer.

The variable effects of immune-humanization in experimental mice that can be attributed to different HSC donors has been discussed at length, and various studies reported that the extent of humanization, as well as the development of individual immune subtypes, can vary from donor to donor [75, 76]. Using different HSC donors, we found similar trends in the timing of human immune cell development, with myeloid cells being the predominant immune cells detected at earlier timepoints post-humanization, while T cells developing at later stages. We also found that E2-induced anemia of immune-humanized mice was consistent across multiple HSC donors. Indeed, the veterinary literature contains several reports of estrogen-mediated BM depression followed by severe anemia in other species including dogs and ferrets [77, 78]. This phenomenon underscores a barrier to the development of immune-humanized models of E2-dependent cancers.

Lastly, the time required for the differentiation of various immune subsets differs and also depends on cytokine signaling. NSG-SGM3 mice are engineered to provide human cytokines needed for myeloid development, but the complex milieu of cytokines and other factors necessary for the development of a complete immune system is still lacking. As proof-of-concept, we noted that we could significantly alter immune cell differentiation by delivering additional exogenous cytokines. Using AAVs as vectors to deliver human TPO, IL-7 and IL-15 to NSG-SGM3 mice in our studies, we found that we could strongly skew immune development towards NK cells at the expense of other cell types (Additional file 1: Fig S6a–f). The addition of cytokines or AAVs did not affect tumor size in two different experiments, and we did not find an effect of matching killer immunoglobulin-like receptors (KIR) between HSC donors and the tumor (Additional file 1: Fig. S6g). This study emphasizes the importance of how a balanced supply of human immune cytokines may be tailored to influence development of immune cell types of interest. Future work will be required to fine-tune physiologically-relevant levels of various cytokines and recapitulate the different immune subtypes that dominate in breast cancer [38].

## Conclusion

Modelling ER+ breast cancer in mice is particularly challenging [47, 48]. While imperfect, we were able to generate an immune-humanized PDX model of ER+ *ESR1*-mutant endocrine-resistant MBC. Given the lack of therapeutic options that these patients face, we hope that our system might provide a model in which to investigate the function of the immune system in the tumor microenvironment of putatively “immune-cold” ER+ breast cancers.

## Supplementary Information

**Additional file 1. Fig. S1**: Estrogen plasma concentrations in NSG or NSG-SGM3 mice bearing HCI-013 PDX tumors receiving either E2 supplemented acidified drinking water (magenta) or implanted with 0.2 mg estrogen pellets (green) (n = 5, see experimental timeline in Fig. 1). Data is normalized to E2 plasma levels of non-tumor bearing NSG mice without exogenous E2 supplementation. **Fig. S2**: Flow cytometry gating strategy for hCD45 as percentage of (a) viable cells and (b) human immune subsets. All gates were set based on FMO controls. **Fig. S3**: List of antibodies used for IHC and IF stains. **Fig. S4**: Quantification workflow of IHC and IF images using QuPath. **Fig. S5**: Weight comparison of estrogen supplemented immune-humanized and non-humanized HCI-013 PDX mice of experiment shown in Fig. 3. All weights are shown relative to the starting weight of each mouse. **Fig. S6**: Utilizing a AAV cocktail as a cytokine delivery system to boost NK cell development. (a) Experimental timeline. AAV-hTPO, AAV-hIL15, AAV-hIL7 were IV injected 4 days prior to HSC implantation. (b-f) Flow cytometry of hCD45 as percentage of (b) viable cells and (c-f) human immune subsets. Injection of AAVs results in reduced human myeloid development (c) but significant increase of human NK cell development (d; n = 2–5). (g) Tumor weights at the experimental endpoint for two independent experiments with KIR-matched (left) or KIR-unmatched HSCs (right) (n = 2–7). Tumors in humanized mice treated with AAV-cytokines didn’t grow significantly different from control groups.(PDF 1049 kb)

## Data Availability

All data generated or analysed during this study are included in this published article [and its supplementary information files].

## Abbreviations

AAV: Adeno-associated virus
ACD: Acid-citrate-dextrose
BM: Bone marrow
CAM5.2: Human-specific cytokeratin
CC3: Cleaved caspase 3
ddPCR: Droplet digital PCR
DMEM/F12: Dulbecco's Modified Eagle Medium/Nutrient Mixture F-12
DMSO: Dimethyl sulfoxide
E2: Estradiol
EI: Estrogen-independent
ER: Estrogen receptor
ESR1: Estrogen receptor 1
FACS: Fluorescence-activated cell sorting
FBS: Fetal bovine serum
FcR: Fc receptor
FFPE: Formalin-fixed paraffin-embedded
FMO: Fluorescence minus one
gDNA: Genomic DNA
GM-CSF: Granulocyte-macrophage colony-stimulating factor
GvHD: Graft-vs-host disease
H: Human
HBEC: Human bronchial epithelial cells
HBSS: Hanks’ balanced salt solution
HE: Hematoxylin/eosin
HER2: Human epidermal growth factor receptor 2
HRP: Horseradish peroxidase
HSC: Hematopoietic stem cell
IF: Immunofluorescense
IHC: Immunohistochemistry
IL-3/7/10/12/15: Interleukin-3/5/10/12/15
IV: Intravenously
KIR: Killer Ig-like receptors
MBC: Metastatic breast cancer
MFP: Mammary fat pad
MISTRG: Mouse strain C;129S4-Rag2tm1.1Flv Csf1tm1(CSF1)Flv Csf2/Il3tm1.1(CSF2,IL3)Flv Thpotm1.1(TPO)Flv Il2rgtm1.1Flv Tg(SIRPA)1Flv/J
Ms: Mouse
NBF: Neutral buffered formalin
NK cell: Natural killer cell
NSG-SGM3: Mouse strain NOD.Cg-Prkdcscid Il2rgtm1Wjl Tg(CMV-IL3,CSF2,KITLG)1Eav/MloySzJ
NSG: Mouse strain NOD.Cg-Prkdcscid Il2rgtm1Wjl/SzJ
OVX: Ovariectomized
PARP: Poly (ADP-ribose) polymeras
PBMC: Peripheral blood mononuclear cell
PBS: Phosphate-buffered saline
PDX: Patient-derived xenograft
PHH3: Phospho-histone H3
PR: Progesterone receptor
RBC: Red blood cell
ROI: Region of interest
SCF: Stem cell factor
SEM: Standard error mean
SERD: Selective estrogen receptor degrader
TAM: Tumor-associated macrophage
TNBC: Triple negative breast cancer
TPO: Thrombopoietin
WT: Wildtype
