# Supplementary material for: An immune-humanized patient-derived xenograft model of estrogen-independent, hormone receptor positive metastatic breast cancer
Source: Breast Cancer Res. 2021 Oct 30;23:100. doi: 10.1186/s13058-021-01476-x (PMC8556932; doi:10.1186/s13058-021-01476-x)
Supplement: Supplementary file 1 — Additional file 1. Fig. S1: Estrogen plasma concentrations in NSG or NSG-SGM3 mice bearing HCI-013 PDX tumors receiving either E2 supplemented acidified drinking water (magenta) or implanted with 0.2 mg estrogen pellets (green) (n = 5, see experimental timeline in Fig. 1). Data is normalized to E2 plasma levels of non-tumor bearing NSG mice without exogenous E2 supplementation. Fig. S2: Flow cytometry gating strategy for hCD45 as percentage of (a) viable cells and (b) human immune subsets. All gates were set based on FMO controls. Fig. S3: List of antibodies used for IHC and IF stains. Fig. S4: Quantification workflow of IHC and IF images using QuPath. Fig. S5: Weight comparison of estrogen supplemented immune-humanized and non-humanized HCI-013 PDX mice of experiment shown in Fig. 3. All weights are shown relative to the starting weight of each mouse. Fig. S6: Utilizing a AAV cocktail as a cytokine delivery system to boost NK cell development. (a) Experimental timeline. AAV-hTPO, AAV-hIL15, AAV-hIL7 were IV injected 4 days prior to HSC implantation. (b-f) Flow cytometry of hCD45 as percentage of (b) viable cells and (c-f) human immune subsets. Injection of AAVs results in reduced human myeloid development (c) but significant increase of human NK cell development (d; n = 2–5). (g) Tumor weights at the experimental endpoint for two independent experiments with KIR-matched (left) or KIR-unmatched HSCs (right) (n = 2–7). Tumors in humanized mice treated with AAV-cytokines didn’t grow significantly different from control groups.(PDF 1049 kb) [file 13058_2021_1476_MOESM1_ESM.pdf]

Suppl. Fig. 1

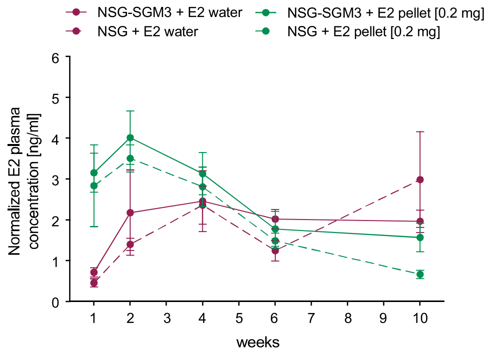

NSG-SGM3 + E2 water
NSG + E2 water
NSG-SGM3 + E2 pellet [0.2 mg]
NSG + E2 pellet [0.2 mg]
Normalized E2 plasma concentration [ng/ml]
0
1
2
3
4
5
6
1
2
3
4
5
6
7
8
9
10
weeks

Suppl. Fig. 2

a

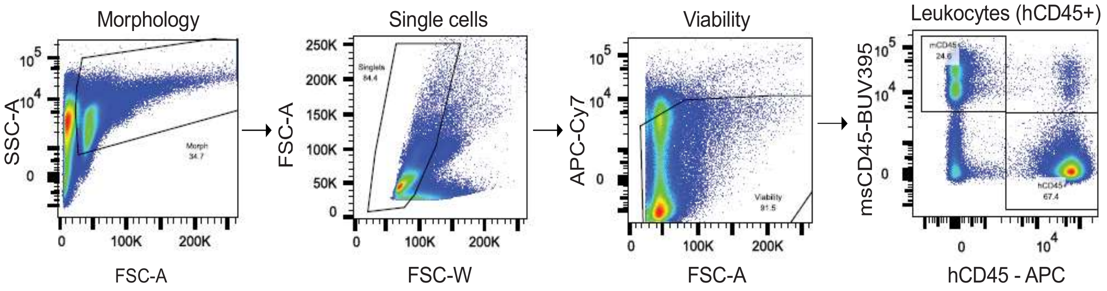

b

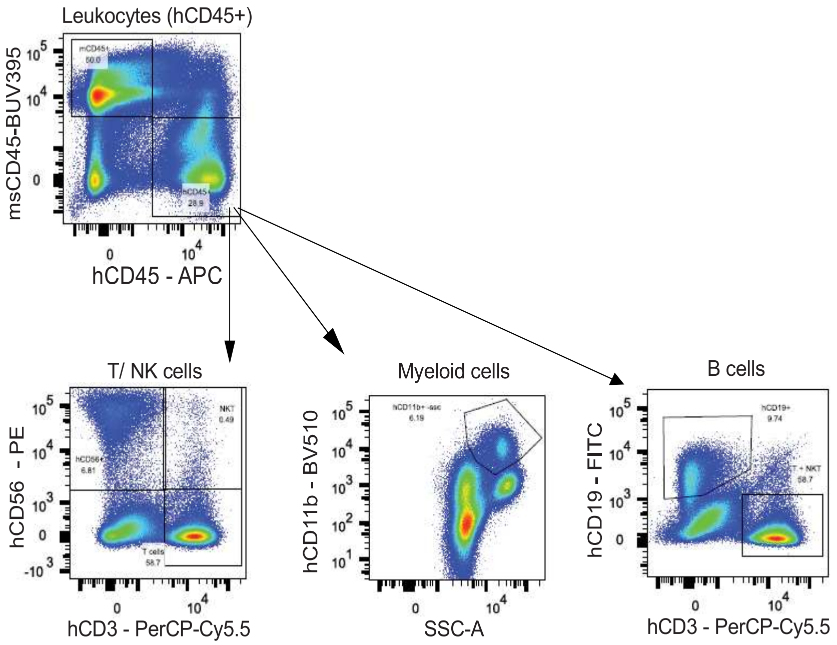

Suppl. Fig. 3

ANTIBODY	METHOD	AR	DILUTION	INCUBATION	VENDOR, CATALOG NUMBER
**Anti-Cytokeratin, clone CAM5.2**	IHC	Trypsin	Neat	1h; RT	BD Biosciences, #349205
**Cleaved Caspase 3 (Asp175), clone 5A1E**	IHC	pH 6	1/150	1h; RT	Cell Signaling Technology, #9664
**CD3, clone LN10**	IHC	pH 9 (T)	1/250	1h; 37C	Leica Biosystems, #NCL-L-CD3-565
**CD20cy, clone L26**	IHC	pH 6	1/200	45’, RT	Agilent Technologies, #M0755
**CD31 (PECAM-1), clone D8V9E**	IHC	pH 6	1/70	1h; RT	Cell Signaling Technology, #77699
**CD45, clone PD7/26 + 2B11**	IHC; IF	pH 6	1/50	1h; RT	Agilent Technologies, #M0701
**CD56 (NCAM), clone CD564**	IHC	pH 10 (T)	1/50	1h; RT	Leica, #NCL-L-CD56-504
**ERa, clone SP1**	IHC	pH 9 (T)	1/35	1h; RT	Thermo Fisher, #MA139540
**Itgam/CD11b, clone D6X1N**	IHC; IF	pH 6	1/50	1h; RT	Cell Signaling Technology, #49420
**Phospho Histone H3 (Ser10)**	IHC	pH 6	1/150	1h; RT	Cell Signaling Technology, #9701
**EnVision+ Single Reagents (HRP. Mouse)**	IHC	-	Ready-to-use	30 min; RT	Agilent Technologies, #K4001
**EnVision+ Single Reagents (HRP. Rabbit)**	IHC	-	Ready-to-use	30 min; RT	Agilent Technologies, #K4003
**Biotinylated IgG Secondary Antibody, Anti-Rb**	IHC; IF	-	1/250	30 min; RT	Vector Labs, #BA-9200
**Biotinylated IgG Secondary Antibody, Anti-Ms**	IHC; IF	-	1/200	30 min; RT	Vector Labs, #BA-1000
**Goat anti-Rb IgG (H+L) Highly Cross-Adsorbed Secondary Antibody, Alexa Fluor 555**	IF	-	1/500	30 min; RT	Thermo Fisher, #A-21429

AR = Antigen Retrieval; IF = Immunofluorescence; IHC = Immunohistochemistry; Ms = Mouse; ON = overnight; pH 6 = Sodium Citrate Buffer; pH 9 = Tris-EDTA Buffer; pH 10 = Tris Buffer; Rb = Rabbit; RT = room temperature; T = Tween 20 (0.05%)

Suppl. Fig. 4

QuPath quantification

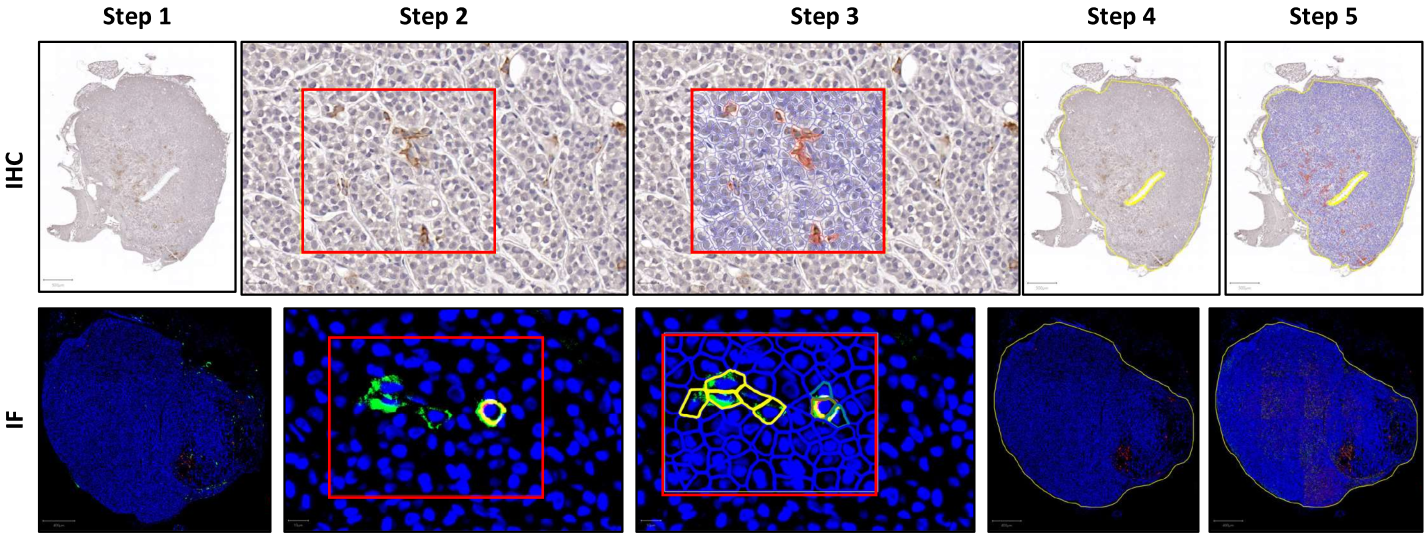

Suppl. Fig. 5

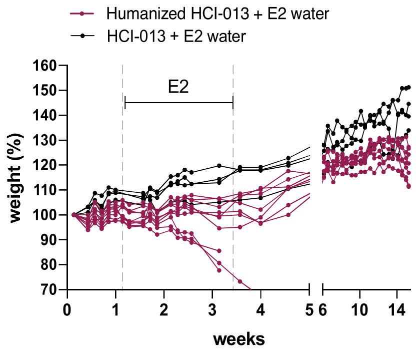

Humanized HCI-013 + E2 water
HCI-013 + E2 water
E2
weight (%)
160
150
140
130
120
110
100
90
80
70
0
1
2
3
4
5
6
10
14
weeks

Suppl. Fig. 6

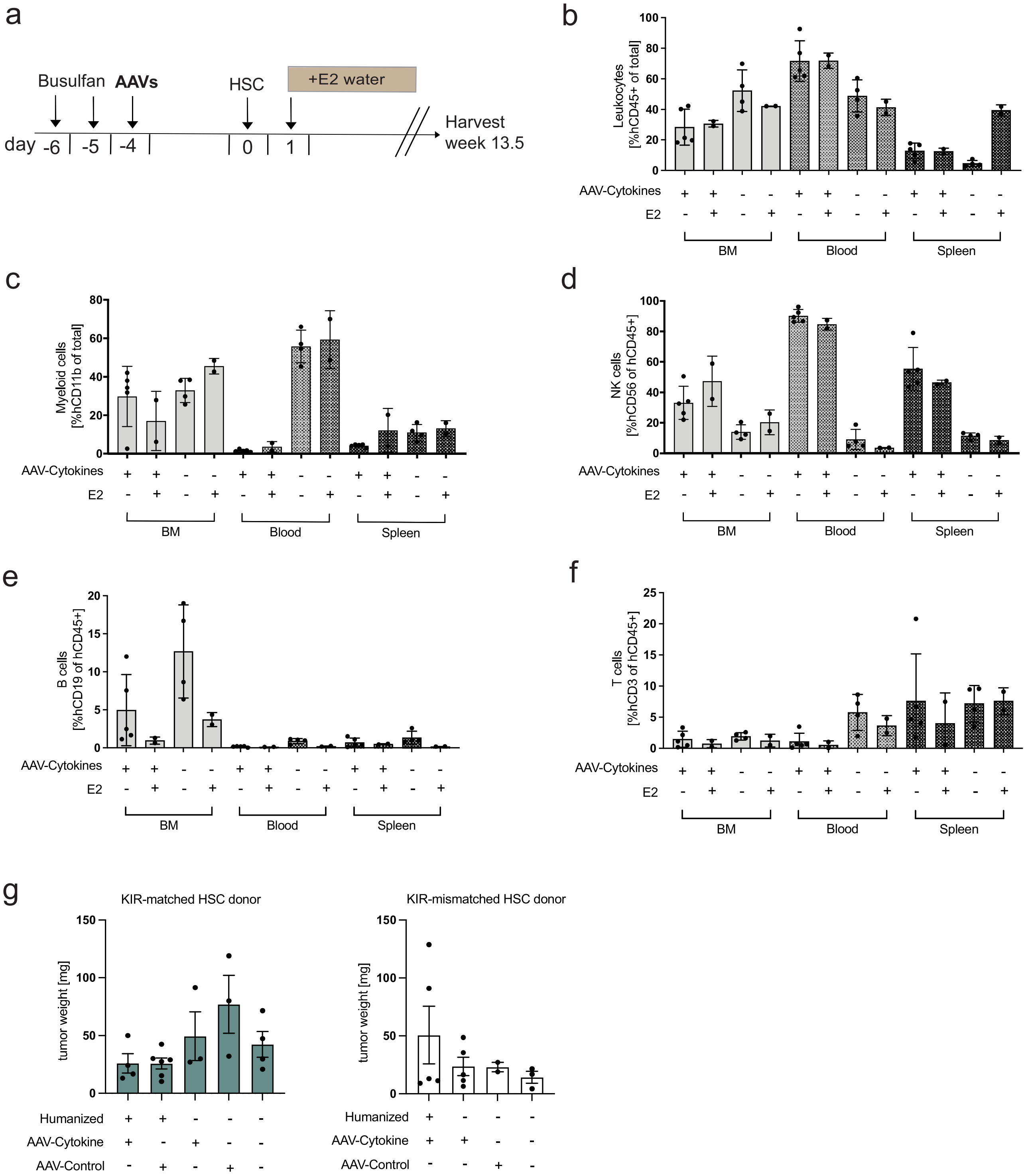
a
Busulfan
AAVs
HSC
+E2 water
day
-6
-5
-4
0
1
Harvest
week 13.5
b
Leukocytes
[%hCD45+ of total]
AAV-Cytokines
E2
BM
Blood
Spleen
c
Myeloid cells
[%hCD11b of total]
AAV-Cytokines
E2
BM
Blood
Spleen
d
NK cells
[%hCD56 of hCD45+]
AAV-Cytokines
E2
BM
Blood
Spleen
e
B cells
[%hCD19 of hCD45+]
AAV-Cytokines
E2
BM
Blood
Spleen
f
T cells
[%hCD3 of hCD45+]
AAV-Cytokines
E2
BM
Blood
Spleen
g
KIR-matched HSC donor
tumor weight [mg]
Humanized
AAV-Cytokine
AAV-Control
KIR-mismatched HSC donor
tumor weight [mg]
Humanized
AAV-Cytokine
AAV-Control
